# Revised version (INFD-D-20-00242): impact of 16S rDNA sequencing on clinical treatment decisions: a single center retrospective study

**DOI:** 10.1186/s12879-021-05892-4

**Published:** 2021-02-18

**Authors:** Axel Ursenbach, Frédéric Schramm, François Séverac, Yves Hansmann, Nicolas Lefebvre, Yvon Ruch, Xavier Argemi

**Affiliations:** 1 Laboratoire de bactériologie, Faculté de Médecine, Université de Strasbourg, 3 rue Koeberlé, 67000 Strasbourg, France; 2 Service de Santé Publique, Hôpitaux Universitaires de Strasbourg, Strasbourg, France; 3 Maladies Infectieuses et Tropicales, Hôpitaux Universitaires de Strasbourg, Strasbourg, France

**Keywords:** 16S PCR, Clinical significance, Polymerase chain reaction, Antibiotic therapy

## Abstract

**Background:**

PCRs targeting 16S ribosomal DNA (16S PCR) followed by Sanger’s sequencing can identify bacteria from normally sterile sites and complement standard analyzes, but they are expensive. We conducted a retrospective study in the Strasbourg University Hospital to assess the clinical impact of 16S PCR sequencing on patients’ treatments according to different sample types.

**Methods:**

From 2014 to 2018, 806 16S PCR samples were processed, and 191 of those were positive.

**Results:**

Overall, the test impacted the treatment of 62 of the 191 patients (32%). The antibiotic treatment was rationalized in 31 patients (50%) and extended in 24 patients (39%), and an invasive procedure was chosen for 7 patients (11%) due to the 16S PCR sequencing results. Positive 16S PCR sequencing results on cerebrospinal fluid (CSF) had a greater impact on patients’ management than positive ones on cardiac valves (*p = 0.044*). The clinical impact of positive 16S PCR sequencing results were significantly higher when blood cultures were negative (*p < 0.001*), and this difference appeared larger when both blood and sample cultures were negative (*p < 0.001*). The diagnostic contribution of 16S PCR was higher in patients with previous antibiotic treatment (*p < 0.001*).

**Conclusion:**

In all, 16S PCR analysis has a significant clinical impact on patient management, particularly for suspected CSF infections, for patients with culture-negative samples and for those with previous antibiotic treatments.

**Supplementary Information:**

The online version contains supplementary material available at 10.1186/s12879-021-05892-4.

## Introduction

Bacterial infections in hospitalized patients can lead to sepsis and death [1], particularly when the diagnosis is delayed [2]. Prompt appropriate antimicrobial therapy can improve patients’ outcomes.

Gram stain results of biological samples can offer a first guidance, but usually, clinicians have to wait for standard culture results for final bacterial species identification with the help of matrix-assisted laser desorption ionization–time-of-flight mass spectrometry (MALDI-TOF MS) and for secondary antimicrobial susceptibility testing. However, standard cultures have limitations regarding fastidious or even non-cultivable bacteria [3], and false negative results can be obtained following antibiotic treatments [4]. Empirical antibiotic therapies present various pitfalls: broad spectrum or prolonged antibiotic treatments increase the risks of drug toxicity [5], of *Clostridioides difficile* infections [6], of opportunistic fungal infections [7] and of multidrug-resistant bacteria selection [8]. Also, erroneous antibiotic therapies due to the lack of bacterial identification preclude patient recovery. Thus, other rapid molecular tests have emerged to improve microbiological diagnoses.

16S rDNA PCR sequencing, a commonly used broad range PCR analysis, has demonstrated its diagnostic performance on various samples: cardiac valves [9–11], abscesses [12], cerebrospinal fluids (CSFs) [13], bone and joint samples [14], sonication fluids [15], pleural fluids [16] and eye samples [17]. Different primers targeting conserved regions of 16S ribosomal DNAs are used to amplify nucleic acids via PCR, followed by sequencing. Variable regions of this gene allow identification to the species level, especially using phylogenetic tree reconstructions [18]. This method has multiple advantages including large implementation in the routine workflow of clinical laboratories and the possibility to detect non-cultivable bacteria or not viable bacteria following antibiotic therapy [3], but it is also expensive.

The clinical impact of this method on the patients’ treatments in real clinical settings has been rarely studied. Our main objective was to assess the impact of positive 16S rDNA PCR analyzes (in various samples) on clinical outcome of patients. The secondary objectives were to evaluate the bacterial identification performance and the effect of a previous antibiotic therapy on the results and to analyze the management of discordant results between 16S PCR and culture identification.

## Methods

### Study design and ethical considerations

This was a single center retrospective study. We included data from all patients with positive 16S PCR results in the Strasbourg University Hospital laboratory from 2014 to 2018. According to the French legislation (Jardé law, N°2016–800), we sought for the non-opposition of all patients. The Strasbourg Hospital Ethic Committee approved this study (reference FC/dossier 2019–27).

### Data collection

We collected all positive 16S PCR results from the microbiological laboratory information system and recovered clinical and laboratory data from the electronic medical records of the relevant patients. We removed all duplicated results and kept a single result per patient. We collected data on the results of blood cultures, the samples’ Gram stains and cultures, the bacteria identified, the presence of previous antibiotic treatments, the clinical impact of the 16S PCR results and the final diagnoses.

We defined the notion of clinical impact of the 16S PCR as a rationalizing of the antibiotic therapy (‘R’, lower dose, shorter or more targeted antibiotic therapy), an extended antibiotic therapy (‘E’, higher dose, longer or extended spectrum antibiotic therapy) or an intervention (‘I’, invasive procedures). Modifications of the patient’s management had to be directly associated with the 16S PCR results based on medical records. We considered previous antibiotic treatments as significant when given for more than 24 h before the sample collection and when being effective against the identified pathogen.

### Sample analyzes

We analyzed blood cultures using the BD BACTEC™ FX instrument (Becton Dickinson). Cardiac valves, bone samples and abscesses were ground in brain heart infusion broth. We used a published sonication protocol [19]. Samples were cultivated using standard media after Gram-stained standard identifications. According to the sample type, the cultures were incubated under both aerobic (35 °C for Columbia agar + 5% sheep blood and Drigalski, 35 °C in 5% CO_2_ for chocolate agar) and anaerobic conditions (Schaedler agar + 5% sheep blood and thioglycolate broth). Joint fluids, bone samples and sonication fluids were also incubated in BD BACTEC™ Peds Plus™ vials, as described [20]. Bacteria were usually identified with MALDI-TOF MS [21] using the Microflex LT coupled to the MALDI Biotyper algorithm (Bruker) as described [22].

### 16S rDNA PCR protocol

16S rDNA PCRs were performed once a week at the request of clinicians or by microbiologists’ initiative and systematically on cardiac valve samples with suspected endocarditis when the sample’s cultures were negative. Briefly, purified DNA was extracted from clinical samples using MagNA Pure (Roche), PCRs were performed with the LightCycler® 2.0 (Roche) instrument with 27F/16S1RRB primers and sequencing reactions were performed using the Sanger method on an ABI 3730 XL system (Applied Biosystems) as described in detail [23]. A microbiologist analyzed the results using the online tool leBIBI^QBPP^ [24] (https://umr5558-bibiserv.univ-lyon1.fr/lebibi/lebibi.cgi) to compare the tested sequence to sequences derived from the International Nucleotide Sequence Database Collaboration (http://www.insdc.org) – including the GenBank® database and EMBL-ENI –, allowing construction of phylogenetic unrooted approximate maximum-likelihood trees and bacterial final identification by using the minimal patristic distance and the nearest reference strain in the tree as the decision criteria. Conclusions were transmitted to the clinicians.

### Statistical analysis

We presented categorical variables as numbers and percentages and performed comparisons between groups using Fisher’s exact tests. In case of significant differences between multiple groups, we carried out pairwise post hoc tests. Adjusted *p*-values were calculated using the ‘Holm’ method to account for multiple comparisons. We considered *p*-values *< 0.05* as statistically significant. We performed all statistical analyses using the R software version 3.5.1 R core team 2018 (Vienna, Austria; https://www.R-project.org/).

## Results

From 2014 to 2018, 835 16S rDNA PCRs were performed in our laboratory, and 197 (24%) were positive with a final bacterial identification. Among these 835 results, we excluded 23 because we could not determine their type of sample precisely and 6 due to incomplete clinical records. In the end, we analyzed data from 191 positive 16S PCR results from the initial 806 tests. Table 1 shows detailed results from Gram stain direct examinations, cultures and 16S rDNA PCR results. In all, 16S PCRs were positive in 80/173 (46%) cardiac valve, 29/71 (41%) abscess, 14/80 (18%) bone, 11/67 (16%) soft tissue, 24/178 (13%) synovial fluid, 13/119 (11%) CSF, 5/27 (19%) pleural fluid, 10/18 (56%) sonication fluid and 5/57 (9%) aqueous humour samples (Table 2).

**Table 1:** Microbiological characteristics of the population

Sample’s culture		+	+	–	–	N/A	Total
Direct examination		+	–	+	–	N/A	
16S PCR		+	+	+	+	–	
Samples	Cardiac valve	7	7	19	47	93	173
	Abscess	2	8	2	17	42	71
	Bone sample	2	4	0	8	66	80
	Soft tissue	3	2	0	6	56	67^a^
	Synovial fluid	2	9	2	11	154	178
	CSF	2	1	5	5	106	119
	Pleural fluid	1	0	1	3	22	27
	Sonication fluid	1	6	2	1	8	18
	Aqueous humour	0	2	1	2	52	57
	Other	0	0	0	0	16	16^b^
Total		20	39	32	100	615	806

^a^23 cerebral biopsies, 15 synovial biopsies, 13 lymph nodes, 3 bone marrows, 4 skin biopsies, 2 vascular biopsies, 1 bladder, 1 oesophageal, 1 hepatic, 1 muscular, 1 subcutaneous, 1 pericardial and 1 ventricular biopsy

^b^12 EDTA blood, 2 pericardial fluids, 1 ascitic fluid and 1 peritoneal dialysate samples

*Abbreviations*: *PCR* polymerase chain reaction, *CSF* cerebrospinal fluid

**Table 2:** Proportion of positive 16S PCR regarding sample types analyzed

Type of sample	16S PCR	Positive 16S PCR (%)
Cardiac valve	173	80 (46%)
Abscess	71	29 (41%)
Bone sample	80	14 (18%)
Soft tissue	67	11 (16%)
Synovial fluid	178	24 (13%)
CSF	119	13 (11%)
Pleural fluid	27	5 (19%)
Sonication fluid	18	10 (56%)
Aqueous humour	57	5 (9%)
Other	16	0
Total	806	191 (24%)

*Abbreviations*: *PCR* polymerase chain reaction, *CSF* cerebrospinal fluid

We found clinical impacts of the 16S PCR results in 62/191 (32%) patients, consisting of 31 (50%) ‘Rationalizing’, 24 (39%) ‘Extended spectrums’ and 7 (11%) ‘Interventions’. Considering all 16S PCR performed in our laboratory during the study period, we estimated the clinical impact to be 8% (62 patients over 806).

In terms of only samples with positive 16S PCR results, the clinical impact differed amongst the various sample types (*p = 0.002*) (Table 3). Our pairwise analysis showed that positive 16S PCR results were more likely to change patients’ treatments if they had CSF samples than if they had cardiac valve samples (*p = 0.044*).

**Table 3:** Efficiency of a positive 16S PCR on the patient’s management regarding sample types analyzed

Type of sample	Positive 16S PCR	Clinical impact	Clinical impact (%)	*p*-value
**Cardiac valve**	**80**	**13**	**16**^**a**^	*0.002*
Abscess	29	13	45	
Bone sample	14	6	43	
Soft tissue	10	2	20	
Synovial fluid	25	11	44	
**CSF**	**13**	**8**	**62**^**a**^	
Pleural fluid	5	3	60	
Sonication fluid	10	4	40	
Aqueous humour	5	2	40	
Total	191	62	32	–

^a^Significant pairwise comparison (*p = 0.044*)

*Abbreviations*: *PCR* polymerase chain reaction, *CSF* cerebrospinal fluid

The patients’ treatments also altered significantly when considering all the 16S PCR results according to sample types (*p = 0.023*) (Table 4), but pairwise analysis was not statistically significant. The clinical impact was highest for patients with abscess samples (18%) and sonication fluid samples (22%), but this population was small (18 patients). Conversely, 16S PCR results on soft tissue and aqueous humour samples were less likely to change the patients’ treatments (2/67 or 3% and 2/57 or 4% of clinical impact, respectively). On synovial fluids, CSF, bone, cardiac valve and pleural fluid samples, the impact of a 16S PCR result was intermediary, ranging from 6 to 11%. We did not find any clinical impact on other sample types. Proportion of ‘Rationalizing’, ‘Extended spectrum’ and ‘Interventions’ regarding sample types is described in Table 4.

**Table 4:** Efficiency of a 16S PCR on the patient’s management regarding sample types analyzed including the type of clinical impact

Type of sample	16S PCR	Clinical impact				*p*-value
		R^a^	E^b^	I^c^	Total	
Cardiac valve	173	6	7	0	13 (8%)	*0.023*
Abscess	71	7	3	3	13 (18%)	
Bone sample	80	4	1	1	6 (8%)	
Soft tissue	67	0	2	0	2 (3%)	
Synovial fluid	178	4	4	3	11 (6%)	
CSF	119	3	5	0	8 (7%)	
Pleural fluid	27	1	2	0	3 (11%)	
Sonication fluid	18	4	0	0	4 (22%)	
Aqueous humour	57	2	0	0	2 (4%)	
Other	16	0	0	0	0	
Total	806	31	24	7	62 (8%)	–

^a^Rationalizing of the antibiotic therapy

^b^Extended antibiotic therapy

^c^Intervention

*Abbreviations*: *PCR* polymerase chain reaction, *CSF* cerebrospinal fluid

Table 5 lists all the pathogens identified by 16S PCR. The most frequently identified pathogens were Gram-positive bacteria 125/203 (62%), but the identification of difficult to grow bacteria was more likely to change a patient’s treatment 12/21 (57%). Bacteria were identified to the species level in 189/203 cases (93%).

**Table 5:** Clinical impact regarding bacteria identified by 16S PCR

Type of bacterium	Identified by 16S PCR	Clinical impact	Clinical impact (%)
Common Gram +	125	29	23
Common Gram −	18	8	44
Anaerobe	39	16	41
Fastidious^a^	21	12	57
Total	203^b^	65	32

^a^containing 9 group HACEK bacteria, 1 *Bartonella* sp., 1 *Bordetella holmesii*, 2 *Nocardia* spp., 1 *Mycobacterium genavense*, 1 *Mycobacterium leprae*, 1 *Neisseria gonorrhoeae*, 2 *Tropheryma whipplei*, 2 *Mycoplasma salivarium* and 1 *Francisella tularensis*

^b^among 191 positive 16S PCR, some were positive for more than one bacterium and 203 bacteria were identified

*Abbreviations*: *PCR* polymerase chain reaction

The proportion of clinical impacts of positive 16S PCR results was significantly superior when blood cultures were negative (52/107, 49%) than when they were positive (10/84, 12%) (*p < 0.001*). This difference was enlarged in cases of simultaneously negative blood culture and sample’s culture, where positive 16S PCR results changed the patients’ treatments in 44/64 (69%) of cases versus changing only 18/127 (14%) of treatment when at least a bacterium had grown (*p < 0.001*).

In patients with previous antibiotic therapy, positive 16S PCR results had a high clinical impact. In this subgroup, 92/118 (78%) samples had negative cultures, whereas only 40/73 (55%) samples had negative cultures in patients without previous efficient antibiotic treatments (*p < 0.001*). We found a clinical impact for the positive 16S PCR results in 33/118 (28%) patients undergoing an efficient antibiotic therapy.

Among 191 positive 16S PCR, blood or sample cultures were positive in 127 cases. The result between PCR and culture was discordant in 38 cases (Table S1). In these situations, 16S PCR results were considered as clinically relevant in 15 cases. For 2 patients, 16S PCR identified an additional bacterium considered by the clinicians. For 7 patients, the bacteria identified by PCR and culture were different and culture was considered as a contamination. For 3 patients, bacteria identified were different and both were considered by clinicians. For 2 patients, the identification was more precise with 16S PCR (i.e. species). For 1 patient, the identification was more precise with 16S PCR, but culture identified one more bacterium considered as a pathogen.

## Discussion

This study showed that 32% of our positive 16S rDNA PCR results (62/191) over 5 years had clinical impacts on the patients’ treatment, and this ratio reached 8% when considering all 16S rDNA PCR tests performed (62/806). Sonication fluid, cardiac valve and abscess samples were more likely to lead to positive 16S DNA PCR results than other sample types.

Few studies have evaluated the global clinical impact of 16S PCR: In 2018, O’Donnell et al. found in a retrospective analysis that 16S PCR results modified patients care in 7 of 49 patients (14%); the antibiotic treatments were narrowed in 5 patients and stopped in 2, particularly in those with neurosurgical samples [25]. In another small patient cohort, Akram et al. found in 2017 that 16S PCR results changed patients’ treatments in 9/32 cases (28%); antibiotic therapies were narrowed in 5 cases and stopped in 2 [26].

Considering patients with cardiac valve samples, we found a clinical impact for 16% of the patients with positive 16S PCR results. Regarding every 16S PCR test, the clinical impact was limited to 8% cases. This result is consistent with those of two other studies where 16S PCR results modified patients’ treatments in 10% [27] and 15% [28] of the patients in a prospective cohort of 127 patients and a retrospective cohort of 46 patients, respectively. Conversely, regarding bone and joint infections, we found that positive 16S PCR results greatly led to patients’ treatment modifications in cases with positive synovial fluid (44%, 11/25), bone (43%, 6/14) and sonication fluid (40%, 4/10). Those may be explained by the frequent negativity of cultures in cases of bone and joint samples than in cases of intravascular infections, highlighting the importance of molecular analyzes like the 16S PCR sequencing. Among the 16S PCR results, the clinical impact for synovial fluid, bone and sonication fluid samples were respectively 6, 8 and 22% and higher than expected by a literature review from Saeed et al., where the 16S PCR results provided a microbiological diagnosis in 141/3840 culture-negative prosthetic joint infections (3.7%) [29]. With regard to abscesses, positive 16S PCR results had clinical impacts in 45% cases, even though this technique is theoretically limited when identifying bacteria from polymicrobial samples [30].

Considering only the positive 16S PCR results, we showed that the clinical impact was significantly higher for patients with CSF samples than for those with cardiac valve samples. This can be explained by the low rate of positive cultures and blood cultures in cases of meningitis and the high frequency of neurosurgical infections that can be tough to diagnose and can involve nosocomial pathogens. On the other side, an empirical antibiotic treatment is often started prior to surgery for endocarditis. The lower clinical impact of a 16S PCR-positive result for the management of endocarditis may also be related to the more frequent positivity of blood cultures before any surgical procedures. As a regional reference center for cardiac surgery [31], we admit numerous patients who have had positive blood cultures in peripheral hospitals. Among 80 positive 16S rDNA PCR on cardiac valves, a blood culture was positive from other peripheral hospitals in 28 cases. Nonetheless, we performed 16S PCR tests systematically in our laboratory for patients with cardiac valves when the sample’s cultures are negative despite previous documentation of infection.

As expected, we found that overall, changes in the patients’ treatments were most frequent for patients with negative culture samples, and this may remain the first indication for 16S PCR tests considering the cost of this time-consuming analysis. This is particularly expected in patients pre-treated with empirical antibiotic therapies or in cases of difficult to grow bacteria. More rarely, 16S PCR tests can be helpful even when blood or culture samples are positive to provide a more accurate species identification producing phylogenetic data.

We are aware of our study’s limitations. This was a single center and retrospective study for which bacteriological data were limited to a specific hospital; thus, potentially relevant biases are unavoidable. Secondly, we did not calculate sensitivity, specificity, or predictive positive and predictive negative values of our 16S PCR tests because the study design did not allow it. Our patients had a high probability of presenting bacterial infections, and we did not have a control group representing true negatives. In our laboratory, 16S PCR is currently performed in every cardiac valve with negative cultures when endocarditis is suspected, despite positive blood cultures, which may introduce a selection bias decreasing its clinical impact compared to other sample types. The turnaround time to result of 16S PCRs was not known.

The 16S rDNA PCR test has several limitations itself. Even if using a broad range PCR, the primers are not universal; the choice in the primers and their degenerate oligonucleotides determines the sensitivity for different species [32]. Studies have described a possible persistence of bacterial DNA (especially in cases with previous endocarditis) that could lead to false-positive results [33, 34]. The test’s interpretation should always consider concomitant clinical data. The analysis of chromatograms from polymicrobial infections can reveal itself difficult using Sanger’s sequencing [30], and samples should always be collected from normally sterile sites only.

## Conclusion

To conclude, 16S PCR is a useful tool for managing patients with infections, particularly when standard cultures are negative and for those with CSF samples, bone and joint infections or abscesses. This tool is probably cost-effective and clinically relevant if patients and samples are carefully selected.

## Supplementary Information

**Additional file 1: Table S1** Management of discordant positive results between 16S PCR and culture

## Data Availability

The data that support the findings of this study are available from the corresponding author, upon request.

## Abbreviations

CSF: Cerebrospinal fluid
DNA: Deoxyribonucleic acid
MALDI-TOF MS: Matrix assisted laser desorption/ionization time of flight mass spectrometry
PCR: Polymerase chain reaction
rDNA: Ribosomal DNA
