# Correction to: Impact of 16S rDNA sequencing on clinical treatment decisions: a single center retrospective study

**DOI:** 10.1186/s12879-021-05938-7

**Published:** 2021-03-18

**Authors:** Axel Ursenbach, Frédéric Schramm, François Séverac, Yves Hansmann, Nicolas Lefebvre, Yvon Ruch, Xavier Argemi

**Affiliations:** 1 Laboratoire de bactériologie, Faculté de Médecine, Université de Strasbourg, 3 rue Koeberlé, 67000 Strasbourg, France; 2 Service de Santé Publique, Hôpitaux Universitaires de Strasbourg, Strasbourg, France; 3 Maladies Infectieuses et Tropicales, Hôpitaux Universitaires de Strasbourg, Strasbourg, France

**Correction to: BMC Infect Dis 21, 190 (2021)**

**https://doi.org/10.1186/s12879-021-05892-4**

After publication of the original article [1], an error was identified in the article title.

The incorrect title is:

Revised version (INFD-D-20-00242): impact of 16S rDNA sequencing on clinical treatment decisions: a single center retrospective study.

The correct title is:

Impact of 16S rDNA sequencing on clinical treatment decisions: a single center retrospective study.

The original article has been corrected.
