# Supplementary material for: Revised version (INFD-D-20-00242): impact of 16S rDNA sequencing on clinical treatment decisions: a single center retrospective study
Source: BMC Infect Dis. 2021 Feb 18;21:190. doi: 10.1186/s12879-021-05892-4 (PMC7890971; doi:10.1186/s12879-021-05892-4)
Supplement: Supplementary file 1 — Additional file 1: Table S1 Management of discordant positive results between 16S PCR and culture [file 12879_2021_5892_MOESM1_ESM.docx]

**Table S1** Management of discordant positive results between 16S PCR and culture

| Type of discordance | Details |
| --- | --- |
| More pathogen identified (14) | **Result of 16S PCR considered (2)** |
|  | Result of blood/sample culture considered (7) |
|  | 16S PCR considered as a contamination (1) |
|  | Blood/sample culture considered as a contamination (4) |
| Different pathogens (23) | 16S PCR considered as a contamination (8) |
|  | **Blood/sample culture considered as a contamination (7)** |
|  | **Identification was more precise with 16S PCR (2)** |
|  | **Results of blood/sample culture and 16S PCR considered (3)** |
|  | Much closed bacteria identified (3) |
| **More pathogen identified by culture but better identification with 16S PCR (1)** | |

Cases where 16S PCR was clinically relevant are in **bold text**

Abbreviation: PCR, polymerase chain reaction
